# Enhanced-Dose Statins for ST-Segment Elevation Myocardial Infarction Patients after Emergency Percutaneous Coronary Intervention

**DOI:** 10.1155/2022/2751750

**Published:** 2022-06-28

**Authors:** Wenzhong Chen, Zhiwen Fan, Canhui Huang, Zhiyuan Han, Junying Liu

**Affiliations:** ^1^Department of Cardiovascular Medicine, Guangzhou First People's Hospital, School of Medicine, South China University of Technology, Guangzhou, Guangdong 510180, China; ^2^Department of Cardiology, The PLA 74th Group Army Hospital, China; ^3^Department of Endocrinology, The First Affiliated Hospital of Shenzhen University, Health Science Center; Shenzhen Second People's Hospital, Shenzhen, China

## Abstract

**Background:**

Acute ST-segment elevation myocardial infarction (STEMI) is a serious multiple acute cardiovascular disease. This study investigated the effect of statins on the efficacy and prognosis of STEMI patients after emergency PCI.

**Methods:**

From October 2019 to January 2021, 98 patients with STEMI in our hospital were selected and divided into study group and control group. The study group took atorvastatin 40 mg 2 hours before surgery, 40 mg/day after surgery, and 20 mg/day 1 week later. The control group received 20 mg of atorvastatin every night after admission. The cardiac output, left ventricular ejection fraction, blood flow classification, vagus nerve function, heart rate deceleration force and chemoreflex sensitivity were compared between the two groups, and recorded the incidence of adverse reactions before and after treatment and 3 months after treatment. The number of major adverse cardiac events (MACEs) was also recorded.

**Results:**

Before treatment, there were no differences in CO, CI, and LVEF between the study and control groups. After treatment, CO, CI, and LVEF in the study group were significantly higher than those in the control group. Before treatment, there was no significant difference in TIMI blood flow classification among the groups, and after treatment, the study group was better than the control group. DC and ChRS were significantly higher in the study group than in the control group. There was no difference in the incidence of adverse reactions between the study group and the control group. However, the incidence of MACE in the study group was lower than that in the control group.

**Conclusion:**

Enhanced-dose atorvastatin for STEMI patients improved PCI treatment effect, cardiac function, and vagus nerve function and reduced the incidence of adverse cardiac events. Thus, statins are safe and worth considering.

## 1. Introduction

Acute ST-segment elevation myocardial infarction (STEMI) is a serious and multiple acute cardiovascular disease. The occurrence of STEMI is mainly due to the rupture, excessive activation, and accumulation of unstable plaques in the coronary arteries, forming clots that block the coronary arteries, leading to myocardial ischemia and irreversible damage to myocardial cells. STEMI poses a serious threat to the lives of patients [1–3]. In recent years, the incidence of STEMI has continuously increased as the population ages and poor lifestyle habits increase. Therefore, the safe and effective treatment of STEMI is crucial [4].

Percutaneous coronary intervention (PCI) can be used to treat STEMI by rebuilding the blood supply and opening the blocked vessels to save ischemic-damaged myocardium [5]. However, some STEMI patients still have abnormal myocardial perfusion, and the damaged myocardium remains unprotected after PCI. Statins have antioxidant effects, reduce inflammation, improve endothelial function, and improve plaque stability. Therefore, the use of statins during treatment can improve the therapeutic effect of PCI. However, the appropriate dose and safety need to be further confirmed [6, 7]. Previous study indicated that atorvastatin therapy increases heart rate variability, decreases QT variability, and shortens QTc interval duration in patients with advanced chronic heart failure [8].

Myocardial reperfusion and vagal function are important clinical indicators to evaluate the clinical treatment and prognosis of patients with cardiovascular disease. However, there are few systematic studies on how booster-dose statins affect vagal function after emergency PCI in STEMI patients [9, 10]. Therefore, we investigated the efficacy of enhanced-dose statins in this patient population following a randomized controlled protocol.

## 2. Materials and Methods

### 2.1. Baseline Data

The protocol of study was approved by the ethics committee of Guangzhou First People's Hospital. From October 2019 to January 2021, 98 patients with STEMI in our hospital were selected and divided into study and control groups using a simple randomized method (49 patients per group). Gender, age, smoking and alcohol consumption, body mass index, and the Killip classification of cardiac function and basic diseases were recorded for comparison between the two groups (Table 1).

### 2.2. Selection Criteria

#### 2.2.1. Inclusion Criteria

Patients were included if they met the STEMI diagnostic criteria [11], serum myocardial necrosis markers increased ≥2 times, there was ST elevation in at least two adjacent leads, a chest lead > 0.2 mV or a pathological Q wave, or a limb lead > 0.1 mV, or they had persistent chest pain ≥ 30 min. Further, all patients received PCI treatment, were confirmed by electrocardiogram, were treated at the first onset, had cardiac function classified as Killip classes I-III, and gave informed consent.

#### 2.2.2. Exclusion Criteria

Patients with other cardiovascular and cerebrovascular diseases, immune system diseases, infectious diseases, renal and hepatic insufficiency who cannot tolerate PCI and drug therapy, received antioxidant or lipid-lowering drug therapy within 1 month before the study, or have allergies were excluded.

### 2.3. Intervention

After admission, both groups received clopidogrel 300-600 mg and aspirin 300 mg. The study group was given atorvastatin 40 mg 2 h before surgery, 40 mg/d after surgery, and 20 mg/d 1 week later [12]. The control group received 20 mg of atorvastatin every night after admission. PCI was performed using the right femoral artery and radial artery puncture, and only the infarct-related vessels were treated. The patient received intravenous heparin (70-100 IU/kg) intraoperatively. 20–25 mL of a platelet glycoprotein II B/III A receptor antagonist or intraventricular injection of 200 *μ*g of sodium nitroso was also administered, and an aortic balloon pulsatile catheter was inserted. Three to 5 days after operation, low molecular weight heparin (4100 U) was subcutaneously injected every 12 h, and long-term oral aspirin (100 mg/d) and clopidogrel (75 mg/d) were administered once.

### 2.4. Observation Indices

Cardiac output (CO), cardiac index (CI), left ventricular ejection fraction (LVEF), and other cardiac function indexes were measured by color Doppler ultrasound (Minrui DC-N2S) before and after treatment in the two groups. The blood flow classification (TIMI) of the two groups before and after treatment was statistically analyzed, and the forward perfusion of the distal occluded vessels was 0. Anterior perfusion of vessels distal to the lesion, but difficulty in filling distal vessels, is grade 1. After at least three cardiac cycles, the distal vessels of the lesion were filled. Contrast can completely fill the distal vessel within three cardiac cycles of grade 3. Vagus nerve function, including heart rate deceleration power (DC) and chemoreflex sensitivity (ChRS), was measured by 24-hour Holter. For ChRS measurements, the patient was in a supine position at rest and inhaled oxygen through a mask for 5 minutes (5 L/min). Venous blood was drawn before and after oxygen inhalation, and venous partial pressure of oxygen (PVO2) was measured. 10 consecutive normal RR intervals were recorded continuously, and the average RR interval was calculated (ChRS = average ΔRR before and after oxygen inhalation/ΔPVO2 before and after oxygen inhalation). The incidence of adverse reactions in the two groups and the incidence of major adverse cardiac events (MACEs) after 3 months were analyzed.

### 2.5. Statistical Method

SPSS 22.0 (IBM Corp., Armonk, N.Y., USA) was used for data analysis. Measurement data were analyzed by *t*-tests and expressed as means ± standard deviation (SD). Enumeration data were analyzed by *χ*^2^ test and expressed as *n* (%). *P* values < 0.05 indicated statistical significance.

## 3. Results

### 3.1. Cardiac Function Index Comparisons

Before treatment, CO, CI, and LVEF did not differ between the groups (CO: 3.32 ± 0.46 vs. 3.19 ± 0.51 L/min, CI: 2.73 ± 0.39 vs. 2.69 ± 0.42 L·min^−1^·m^−2^, and LVEF: 37.79 ± 4.21 vs. 39.05 ± 3.97%; *P* > 0.05). After treatment, CO, CI, and LVEF were significantly higher in the study group than in the control group (CO: 4.69 ± 0.37 vs. 4.18 ± 0.40 L/min, CI: 3.78 ± 0.29 vs. 3.43 ± 0.32 L·min^−1^·m^−2^, and LVEF: 54.48 ± 4.10 vs. 49.98 ± 3.82%; *P* < 0.05; Table 2).

### 3.2. TIMI Blood Flow Grading Comparison

TIMI blood flow grading did not differ between the two groups before treatment (*P* > 0.05). However, it was significantly better in the study group than in the control group after treatment (*P* < 0.05; Table 3).

### 3.3. Vagus Nerve Function Indicator Comparisons

DC and ChRS were significantly higher in the study group than in the control group (DC: 2.53 ± 1.43 vs. 1.98 ± 0.97 ms and ChRS: 3.11 ± 1.04 vs. 1.91 ± 0.89 ms/mm Hg; *P* < 0.05; Table 4).

### 3.4. Comparison of Incidence of Adverse Reactions

The rate of incidence of adverse reactions did not differ between the study group (10.20%) and the control group (6.12%) (*P* > 0.05; Table 5).

### 3.5. Comparison of Incidence of MACEs

The rate of incidence of MACEs was significantly lower in the study group (6.12%) than in the control group (20.41%) (*P* < 0.05; Table 6).

## 4. Discussion

Emergency PCI is an important STEMI treatment as it can completely, quickly, and permanently restore coronary artery blood flow, reduce disease mortality, relieve clinical symptoms, and prolong patients' lives [13]. However, mechanical damage can occur during PCI treatment, resulting in a vascular endothelial tear. Thus, a large amount of subcutaneous tissue is exposed to blood, which can trigger an inflammatory response, damage myocardial cells, activate coagulation functions, and increase the risk of MACE [14, 15]. Previously, dual antiplatelet and antithrombotic interventions mainly focused on perioperative PCI. However, clinical studies indicate that atorvastatin administration before and after an emergency or elective PCI helped reduce the risk of perioperative and postoperative MACE for patients with myocardial infarction [16, 17].

The dose of atorvastatin was controversial, and previous studies indicated that a high-dose loading of statins (80 mg/day) before PCI in patients with ACS reduces MACCE and reduces the risk of MI with no impact on mortality at 30 days [18]. But high-dose atorvastatin pretreatment does not seem to prevent CIN in patients receiving primary angioplasty [19]. In addition, High-dose atorvastatin may produce an optimal result for STEMI patients undergoing PCI by improving microvascular myocardial perfusion [20]. This study investigated the value of atorvastatin as an adjuvant therapy at different doses during PCI treatment for STEMI patients. We found that CO, CI, and LVEF were higher, the TIMI blood grade was better, and the MACE incidence was lower in the study group than in the control group (*P* < 0.05). The incidence of adverse reactions did not differ between the two groups, suggesting that, compared with routine dosing of atorvastatin, increasing the atorvastatin dosage improved the therapeutic effect of PCI in STEMI patients, restored cardiac function and blood flow grading, and helped reduce the risk of MACE. These results are noteworthy as they suggest a good disease outcome without an increased risk of adverse reactions, and that the treatment is safe. This may be because atorvastatin has a unique chemical structure, namely, aromatic groups, which form hydroxyl-activated metabolites that prolong the half-life of atorvastatin to improve vascular endothelial function, reduce cholesterol, and inhibit platelet aggregation, oxidation, and inflammation. They also increase other cardiovascular protective functions and regulate lipids, which can be further improved by enhanced-dose application [21, 22]. For example, an enhanced atorvastatin dose reduces platelet aggregation and activation. Further, there are different degrees of inflammatory responses in STEMI patients that cause myocardial injury, elicit an immune response, and increase the risk of ventricular muscle remodeling, which worsens cardiac function. Enhanced doses of atorvastatin can minimize these reactions through a potent anti-inflammatory effect [23].

In the early stage of STEMI, cardiac autonomic nerve necrosis, injury, and remodeling occur, mainly manifesting as sympathetic nerve remodeling and vagus nerve function decline. The stability of myocardial cell electrical activity decreases, and the myocardial metabolism is abnormal, leading to poor phenomena such as pleomorphism, persistent ventricular tachycardia, ventricular fibrillation, and eventually sudden cardiac death. Foreign studies indicate that abnormal vagus nerve function and sympathetic nerve remodeling are the main causes of myocardial excitability enhancement and abnormal conduction and are important electrophysiological mechanisms for the occurrence and maintenance of malignant arrhythmias. Autonomic nerve function, especially the vagus nerve state, can predict the occurrence of adverse cardiovascular events. DC has been an important part of Holter electrocardiogram research in recent years. By analyzing the overall 24 h heart rate trend and measuring the deceleration ability, the vagal tone can be quantitatively assessed, allowing for a new noninvasive electrocardiogram technology for screening and warning high-risk MACE patients. ChRS is also a noninvasive indicator reflecting autonomic nerve function and is an independent predictor of adverse cardiovascular events after PCI in STEMI patients. It is better than ventricular arrhythmia, ventricular late potential, and LVEF and can accurately reflect the stability of myocardial electrical activity [24, 25]. However, in our study, DC and ChRS were higher in the study group than in the control group after treatment (*P* < 0.05), further verifying that the enhanced atorvastatin dose is advantageous for PCI in STEMI patients and improves the therapeutic effect and disease prognosis.

In conclusion, intervention with an enhanced atorvastatin dose for STEMI patients improved PCI treatment effect, cardiac function, and vagus nerve function and reduced the incidences of adverse cardiac events. Thus, enhanced statin treatment is safe and worth considering. However, in our study, we did not investigate the long-term living conditions of patients. Therefore, the effect of an enhanced atorvastatin dose on the long-term prognosis of STEMI patients after PCI requires further investigation.

## Figures and Tables

**Table 1:** Comparison of baseline data.

Clinical data	The study group (*n* = 49)	The control group (*n* = 49)	*t*/*χ*^2^	*P*
Age (years old)	44~79 (61.71 ± 12.29)	45~76 (60.28 ± 11.68)	0.590	0.556
Gender				
Male	29 (59.18)	33 (67.35)	0.703	0.402
Female	20 (40.82)	16 (32.65)		
BMI (kg/m^2^)	18.2~27.5 (22.89 ± 3.05)	17.4~28.1 (23.15 ± 2.94)	0.430	0.668
Smoking				
Yes	18 (36.73)	21 (42.86)	0.383	0.536
No	31 (63.27)	28 (57.14)		
Drinking				
Yes	27 (55.10)	24 (48.98)	0.368	0.544
No	22 (44.90)	25 (51.02)		
Basic disease				
Hypertension	15 (30.61)	19 (38.78)	0.721	0.396
Diabetes	13 (26.53)	11 (22.45)	0.221	0.638
Others	5 (10.20)	3 (6.12)	0.136	0.712
Killip grading of cardiac function				
Grade I	17 (34.69)	14 (28.57)	0.494	0.781
Grade II	22 (44.90)	23 (46.94)		
Grade III	10 (20.41)	12 (24.49)		

**Table 2:** Comparison of cardiac function index (mean ± standard deviation).

Time	Groups	Cases	CO (L/min)	CI (L·min^−1^·m^−2^)	LVEF (%)
Before treatment	The study group	49	3.32 ± 0.46	2.73 ± 0.39	37.79 ± 4.21
	The control group	49	3.19 ± 0.51	2.69 ± 0.42	39.05 ± 3.97
	*t* value		1.325	0.489	1.524
	*P* value		0.188	0.626	0.131
After treatment	The study group	49	4.69 ± 0.37	3.78 ± 0.29	54.48 ± 4.10
	The control group	49	4.18 ± 0.40	3.43 ± 0.32	49.98 ± 3.82
	*t* value		6.552	5.673	5.621
	*P* value		0.001	0.001	0.001

**Table 3:** Comparison of blood flow grade (TIMI) (*n* (%)).

Time	Groups	Cases	Grade 0	Grade 1	Grade 2	Grade 3
Before treatment	The study group	49	28 (57.14)	19 (38.78)	2 (4.08)	0 (0.00)
	The control group	49	25 (51.02)	23 (46.94)	1 (2.04)	0 (0.00)
	*χ* ^2^ value		1.092			
	*P* value		0.579			
After treatment	The study group	49	0 (0.00)	0 (0.00)	3 (6.12)	46 (93.88)
	The control group	49	0 (0.00)	2 (4.08)	10 (20.41)	37 (75.51)
	*χ* ^2^ value		7.202			
	*P* value		0.027			

**Table 4:** Comparison of vagus nerve function indicator (mean ± standard deviation).

Groups	Cases	DC (ms)	ChRS (ms/mmHg)
The study group	49	2.53 ± 1.43	3.11 ± 1.04
The control group	49	1.98 ± 0.97	1.91 ± 0.89
*t* value		2.228	6.137
*P* value		0.028	0.001

**Table 5:** Comparison of incidence of adverse reactions (*n* (%)).

Groups	Cases	Abnormal kidney and liver function	Fatigue	Digestive tract reaction	Allergy	The total incidence rate
Study	49	1 (2.04)	2 (4.08)	1 (2.04)	1 (2.04)	5 (10.20)
Control	49	1 (2.04)	0 (0.00)	2 (4.08)	0 (0.00)	3 (6.12)
*χ* ^2^ value						0.136
*P* value						0.712

**Table 6:** Comparison of major adverse cardiac event (*n* (%)).

Groups	Cases	Target vessel reconstruction	Cardiogenic shock	Heart failure	Arrhythmia	Recurrent myocardial infarction	The total incidence rate
Study	49	1 (2.04)	0 (0.00)	1 (2.04)	1 (2.04)	0 (0.00)	3 (6.12)
Control	49	2 (4.08)	1 (2.04)	3 (6.12)	2 (4.08)	2 (4.08)	10 (20.41)
*χ* ^2^ value							4.346
*P* value							0.037

## Data Availability

All data was provided in the manuscript.
